# The Metropolitan Hospital Sunday Fund

**Published:** 1913-12-27

**Authors:** 


### METROPOLITAN HOSPITAL SUNDAY FUND.

### The Discussion at the Annual Meeting.

THE annual meeting of the constituents of the above Fund was held at the Mansion House on Friday, December 19, Alderman Sir John C. Bell in the chair.

The CHAIRMAN said he was sure they would all regret to hear that the Lord Mayor was unable to be present on that occasion, owing to a very pressing engagement.

The SECRETARY (Mr. Arnold James) read the notice convening the meeting, and announced the receipt of letters of regret from the Bishop of Islington and Sir Henry Burdett. He also read the minutes of the last annual meeting and of special meetings held on February 10 and June 20, 1913, respectively, all of which were duly confirmed and signed.

The CHAIRMAN said he presumed that the meeting would take the annual report as read. He also announced that the Council, on the recommendation of the General Purposes Committee, had unanimously resolved that the practice of borrowing convalescent letters in advance—*i.e.*, on account of future grants—be absolutely discontinued, and a public statement of such resolution made at the present meeting.

### A SERIOUS DECREASE IN INCOME.

The Rev. Canon PEARCE, M.A., in moving the adoption of the report of the Council for the year 1913, said that there were two principal things which he desired to bring to the notice of the constituents in regard to this year's report; first of all, the finance, and, secondly, the constitution. With regard to finance, they would see that there was a decrease in receipts of £2,981 as compared with last year. And when they went into it in detail they would find that that decrease was practically equivalent to the decrease in the total of the collections for the Fund in places of worship on Hospital Sunday. It was a serious decrease. It was one which must make them all consider the position in which they stood—namely, that there should be so large a falling off in the total collected, with a probable proportionate falling off of the people present in church, in one single year. But they would also notice, if they looked at the column upon page 10, that there had been a certain changing about in the totals of the last two years, and that while the total of the Sunday collections—the amounts received from congregations—appeared to have fallen from £35,000 to £28,000, on the other hand, the amount received by way of legacies and donations appeared to have risen from £32,000 to £36,500. The fact was explained in the report, and it was just a small matter worthy of notice. During the life of the late Mr. George Herring—during his latter years, at any rate—he was accustomed to contribute to the extent of 25 per cent. of the collections received in the churches, and there was a sort of convention by which all the donations sent to the office of the Fund at the time of Hospital Sunday week were added to the collections at St. Paul's Cathedral, in order that they might receive Mr. Herring's increment. He need not say that Mr. Herring was a consenting party to what might otherwise have been a rather curious arrangement. (Laughter.) But Mr. Herring had passed away; Mr. Herring's gifts now took another form; and it was rather a hint to them that the total of the interest of Mr. Herring's bequest was practically about equal to the total which they themselves raised in the Sunday collections. That was rather a sad reflection, but it was there. This year, then, they had returned to the natural arrangement. The collection attributed to St. Paul's Cathedral in the list on page 8 was the actual collection there taken, and the £4,000 or so received in donations had gone to swell the other column. He would also draw their attention, without discussing it further, to the reprint of the new constitution, which would be found embodied in the report. The mere aspect of that meeting would, he hoped, serve to show them that the Council had, in the proposals which they had put before them for that new constitution, an object which already in the light of that meeting they had very largely attained. The purpose was to keep the Council in touch with a larger proportion of the actual donors to the Hospital Sunday Fund. In other words, where churches were able to send the Fund a large sum they sent more constituents to the annual meeting, not because the Council had a higher regard for the church which sent a large sum than for the church which could only send a smaller sum, but because in the church which sent the large sum the chances were that there was a larger number of contributors who ought to be in touch with the Fund. The numbers present at that meeting was proof, if proof were needed, that the Council had so far succeeded.

The Right Hon. Sir SAVILE CROSSLEY, Bart., P.C., seconding, drew attention to the fact that for the first time for some years past they now had tabulated in an accurate form the collections made in churches. He wished also to point out that, notwithstanding the falling off of the church collections, more money had been distributed this year than ever before. That was due to the Finance Committee, to whom he thought the very greatest credit was due, and he was sure he was only expressing the views of every man and every lady present when he said they were most sincerely indebted to the Finance Committee for the judicious management they had displayed in dealing with their finances. They would notice also this paragraph in the report: "The Council report with satisfaction that the transfer of the residue of Mr. George Herring's estate is now complete." In these times they had to fight, in collecting for the hospitals, a very hard battle. Many people were ready and anxious to ride off on any excuse from the duty of giving, and perhaps just now they had a better excuse than ever before by reason of recent legislation. In saying that he was speaking quite without party feeling, but there was no doubt a sort of excuse had been afforded in that way. The mover of the resolution had pointed out that they were obtaining very much larger sums than ever before from legacies, and he would like to take that opportunity of saying that it must be a great advantage, as things were at present, to the Government to have bequests made rather than gifts during the donor's lifetime to large charities of this sort; and, on the other hand, in his humble opinion, there was very much to be said on the other side, from the point of view of the families of benefactors, or possible benefactors, of such funds as theirs, in favour of the giving away of charitable gifts during their own lifetime. (Applause.)

The resolution was then put to the meeting and carried unanimously.

The Rev. W. HARDY HARWOOD, in moving the election of the retiring members of the Council for the year 1914, said that as that was the first time such a resolution as he was about to propose had been moved, it having been rendered necessary by the new constitution, he might perhaps be allowed to say a sentence or two as one of the sub-committee which had had to draw up that constitution. He wished to say, on their behalf and on behalf of the Council, how grateful they were to the churches for taking up so readily the new methods which had been submitted to them. (Hear, hear.) He believed nearly 90 per cent. of the churches had responded, and responded very quickly, to the appeal that they would send in their reports for the whole year, and not in the somewhat haphazard fashion which had formerly been in vogue. The Bishop of Hull (Rev Prebendary Gurdon), the Dean of Rochester (Rev. Prebendary Storrs), and Mr. James Andrews were not seeking re-election, and he therefore begged to move that the vacancies be filled by the Very Rev. the Chief Rabbi, Mgr. Carton de Wiart, and Sir David Burnett, Bart., and that the remaining members in the list which he had read be re-elected. He ought perhaps to explain that there was one slight variation from the printed notice sent out to the constituents. Since the date of that notice Mgr. Howlett had wished to retire, and the Council therefore now suggested that Mgr. Wiart, who had actually been nominated, but had withdrawn in favour of the Chief Rabbi, should be put on the Council, as representing the Roman Catholic Church, in place of Mgr. Howlett. (Hear, hear.)

The Rev. Prebendary Reynolds briefly seconded the resolution, which was then put and carried unanimously.

Don't Grow Weary but Work for the Hospitals.

The Rev. E. S. Hilliard next moved: "That June 14 be fixed for Hospital Sunday in the year 1914, and that the cordial co-operation of all ministers of religion within the Metropolitan area be again invited in the usual way." To his brother ministers of religion he would like to suggest that they ought to notice very seriously the deficit of £3,000 on last year's collections. His own belief was that the contributions of any congregation very large represented the trouble taken by the minister over and above the mere preaching of the sermons. The trouble he took to prepare the way beforehand, by personal communications or by whatever methods seemed to him best in dealing with his own people—those were the things which very largely represented the differences in the results. Of course, that was not so in regard to all congregations, but he was speaking of many congregations where, if the ministers of religion could be persuaded not to get tired of the cause of the hospitals because they had pleaded it so often, but to keep themselves fresh and to show ingenuity and fertility of resource in trying to induce their people to pay special attention to Hospital Sunday, a great deal could be done to defeat the ravages caused by the week-end, the motor-car, and the general neglect of religious duties. Then to those who belonged to the Church to which he had the honour to belong, especially to those in that diocese, he would say they had got this Juggernaut car of the Archbishops' financial scheme approaching, and he could not see any consideration for the hospitals in anything connected with that financial scheme which he had read or heard as yet. And he did hope that the English Church world would take very good care that whatever else happened under this Juggernaut-car scheme—for he would not deny that he regarded it with profound suspicion, together with a good deal of timidity—(laughter)—they would not allow the hospitals to suffer in any way if they could possibly prevent it. (Applause.) And, lastly, there was one man whom they ought all to try to get at in order that they might, if need be with a sledge-hammer, get it into his head that June 14 was the date of the next Hospital Sunday. They all knew the man he meant, but nevertheless he would just tell them exactly who the man was. There was a very rich man who gave away very little indeed. This man had a little body of candid friends, and they determined to deal as faithfully with him as they could. And so they deputed one of their number to "go for" him quite straight. And he went and said to him, "Now, sir, I think it is true, is it not, that you are very rich indeed?" He said, "Yes, that is quite true." "And I think it is also true that you give away very little indeed?" He replied, "Yes, that is quite true; but, my dear sir, if you only knew how it hurts me to give that, you would wonder that I gave anything at all!" (Laughter.) Now that was the gentleman they had to get at. He was all about, and there was plenty of him—(laughter)—and therefore they would not have any difficulty whatever in finding him. (Applause.)

Sir Ernest Tritton, Bart., who seconded the resolution, said the matter of the collections in the churches was one which came very much and very prominently before his individual attention, because as Chairman of the Finance Committee all the cheques came before him for his endorsement, and in looking over them as he did he was struck by the great liberality this last year of a large number of their churches. He himself believed that, all things considered—times bad, extra taxation, and all the rest of it—the churches did uncommonly well last June in their collections, and, having said that, he would only add that if he were alive and spared to examine the cheques again and endorse them when the next collection came round, he hoped they would give their Chairman of the Finance Committee the opportunity of being still more cheerful and joyful by permitting him to see a still larger collection.

The resolution was carried unanimously.

The Rev. Richard Free (vicar of St. Clement's, Fulham) asked permission to move a resolution which, he said, he had handed to the Chairman.

The Chairman reminded the speaker of Law VI. (*c*), which provided that resolutions subsequent to the ordinary business must be in the form of recommendations to the Council, and asked whether Mr. Free wished the resolution put to the meeting in that form.

The Rev. Richard Free: Certainly; put it to the meeting.

The Chairman then read the resolution, which was as follows: "That no grant be given to hospitals having vivisectional laboratories attached to their medical schools, the practice of vivisection being diametrically opposed to the teaching of Christ." He said it would be necessary for the resolution to be seconded, and if carried it would, of course, go to the Council for consideration.

The Rev. D. G. Camus (St. Aldhelm's, Upper Edmonton) seconded the resolution.

The Chairman then put the resolution to the meeting, and said that in doing so he wished to remind them that the Council were a very busy body of men, with a great deal of work to do. Having said that, he would leave it to the meeting to decide whether this resolution ought to be forwarded to the Council for consideration.

The resolution was declared, on a show of hands, to be lost by a large majority.

The Rev. Canon Rhodes Bristow, M.A., proposed a hearty vote of thanks to the Chairman for presiding, which was carried by acclamation.

